# Mechanical Behavior of Masonry Mortars Made with Recycled Mortar Aggregate

**DOI:** 10.3390/ma13102373

**Published:** 2020-05-21

**Authors:** René Sebastián Mora-Ortiz, Emmanuel Munguía-Balvanera, Sergio Alberto Díaz, Francisco Magaña-Hernández, Ebelia Del Angel-Meraz, Álvaro Bolaina-Juárez

**Affiliations:** División Académica de Ingeniería y Arquitectura (DAIA-UJAT), Universidad Juárez Autónoma de Tabasco, Carretera Cunduacán-Jalpa de Méndez km. 1, Cunduacán, Tabasco CP 86690, Mexico; balvanerae@hotmail.com (E.M.-B.); alberto.diaz@ujat.mx (S.A.D.); francisco.magana@ujat.mx (F.M.-H.); ebelia.delangel@ujat.mx (E.D.A.-M.); aalvarado914130@gmail.com (Á.B.-J.)

**Keywords:** construction and demolition waste, recycled fine aggregate, mortars, sustainable construction, recycled aggregates

## Abstract

Recycling is an important habit to avoid waste. This paper evaluates the performance of masonry mortar, elaborated by replacing natural sand with recycled fine aggregate (RFA) obtained from mortar. Five families of mixtures were prepared with different replacement proportions: 20%, 40%, 60%, and 100%. A 1:4 volumetric cement-to-aggregate ratio was used for all mixtures by experimentally adjusting the amount of water to achieve the same consistency of 175 ± 5 mm. The effects of the following procedures were analyzed: (1) the use of a deconstruction technique to collect the RFA, (2) pre-wetting of the aggregates, and (3) the use of a commercial plasticizer. Experimental results show that it is possible to use this type of recycled fine aggregate as a substitute for natural sand by up to 60% in the manufacture of masonry mortar without significantly affecting its properties.

## 1. Introduction

A guideline for developing new construction materials is to improve material performance, optimize supplies and reduce manufacturing costs. Therefore, it is necessary to develop new techniques and take advantage of materials which are considered waste in this and other industries. For decades, the amount of construction and demolition waste (CDW) has been increasing globally, becoming one of the main agents of environmental pollution.

Using the CDW produced during the demolition of concrete structures as a substitute for thick and fine aggregates in masonry mortar mixtures reduces the amount of pollutant waste released to the environment, compensates for the lack of stone aggregates, and represents an innovation in the development of construction materials [1,2,3]. Recycled aggregates (RA) which are used in replacement of natural gravel, are known as recycled thick aggregates (RTA), whereas RA used to replace sand are called recycled fine aggregates (RFA). RFA may be classified into two types: those obtained from concrete, and those obtained from other materials, such as mortar or ceramic.

The use of RA, especially RFAs obtained from mortar elements, has some drawbacks, most of them associated with the nature of these materials, such as their porosity, their high-water absorption potential, and the possibility of containing pollutants. For these reasons, CDW is not recycled as it should be, which causes its accumulation in landfills, generating pollution. This research presents two possible alternatives for the reuse of mortar RFA as a substitute for sand in new mixtures. The analysis was divided into two stages: first, the characteristics of RAs obtained by a “deconstruction” procedure were compared against those obtained through a conventional demolition. In the second stage, two possible scenarios were analyzed: (i) pre-wetting the RFA before mixing, and (ii) using a commercial plasticizer to reduce the amount of water in the mixture. The performance of mortars made with RFA was compared against a conventional mortar mixture (cement, natural sand, and water).

Currently, several researchers support the use of RTA obtained from the demolition of concrete elements as a partial substitute for natural gravel in the preparation of concrete mixtures [4,5,6]. However, they do not recommend the replacement of natural sand by RFA, because they consider this substitution detrimental to the mechanical properties and durability of concrete [4,7]. Notwithstanding, some researchers, such as Pereira et al. [8], Mefteh et al. [9], Evangelista and de Brito [10], and Cartuxo et al. [11] believe that natural sand can be replaced by RFA in a proportion of up to 20% without significantly affecting the mechanical properties of the concrete. Using RFA as a substitute for natural sand in masonry mortar mixes is the best option, because it has fewer structural requirements than concrete. RFA obtained from concrete elements have been studied by researchers such as Braga et al. [12], Neno et al. [13], Saiz-Martínez et al. [14] and Ng and Engelsen [15], who have proven their viability as a partial substitute for natural sand. However, research on the use of RFA obtained from mortar elements is scarce, due to the inferior physical, mechanical and chemical characteristics of this type of material, such as its high-water absorption potential, porosity, and susceptibility to contain contaminants [16,17]. This has resulted in the accumulation of debris from the demolition of prefabricated mortar elements in sanitary landfills and clandestine landfills, in the over overexploitation of river or quarry sandbanks, and in the increase of energy consumption and CO_2_ emissions as a consequence of crushing rocks to produce fine aggregates [11]. Silva et al. [18] showed that incorporating 10% ceramic RFA improves most of the mortar´s properties. In the research conducted by Jiménez et al. [19], natural sand was replaced by RFA composed by 54% ceramic and 40% masonry mortar. Experimental results showed that replacing natural sand with this type of RFA in a proportion of 40% does not significantly affect mortar properties in fresh and hardened state. On the other hand, Silva et al. [20] demonstrated the technical feasibility of recycling RFA resulting from the demolition of bricks or red clay tiles. They concluded that mortars made with replacement ratios of at least 20% generally show a better performance than conventional mortar, giving emphasis to aspects such as flexural and compressive strengths.

Recycled mortar properties depend on RFA quality, the substitution rate of natural sand, cement content, and water-cement (W/C) ratio. Among these factors, RFA quality is perhaps the most important. It is known that RAs usually contain a certain number of sulfates, chlorides, and other contaminants [21]. These impurities are, for the most part, the product of a lack of demolition planning. Since the reuse of demolition rubble is not considered from the beginning, it is deposited outdoors, or in places where it may be contaminated, allowing its mixture with other materials. Researchers such as Rahal [22], Cachim [23], and Debieb et al. [24] (just to mention a few), have studied the effect of the presence of these impurities on the behavior of concrete which has been manufactured with replacement of natural aggregates by RA. The content of impurities and contaminants in RA can be greatly reduced by means of selective demolition techniques [25,26]. Kumbhar et al. [27], as well as Coelho and de Brito [28], describe deconstruction techniques for obtaining good quality CDW.

Water absorption potential is a determining factor in RFA quality. This is because, in the RA, mortar is attached to the natural aggregate [29,30]. The implication of this RA characteristic is the W/C reduction ratio in the cement paste, which results in poor workability, a greater number of pores, less compression resistance, as well as drying contractions [31,32]. Therefore, to guarantee the workability of the cement paste there are two possible solutions: the incorporation of plasticizers in the mixture [7,11,33,34] or pre-wetting RAs before mixing [9,35,36,37]. Pereira et al. [8] conducted one of the first studies on the effect of superplasticizers on the properties of fresh and hardened concrete made with concrete RFA. Together with Cartuxo et al. [11] and Barbudo et al. [38], they showed that in mixtures in which sand is replaced by RFA, the use of plasticizers improves the mechanical characteristics of concrete. Zega and Maio [39] concluded that using a water-reducing additive produces recycled concrete with adequate performance, which follows the specifications established by different international construction standards.

Studies such as those directed by González et al. [35], Mefteh et al. [9], and Cuenca-Moyano et al. [40], have reported the benefits of pre-wetting RAs before making concrete mixtures. In all of these cases, humidity levels below 100% of absorption capacity were recommended. Researchers like Cabral et al. [41] and Zhao et al. [42] point out that the best results are obtained with humidities lesser than or equal to 80% of absorption capacity.

The main objective of this research was to separately analyze the influence of pre-wetting RFAs and using commercial plasticizers during the creation of new masonry mortars with partial substitution of natural sand by RFA obtained from mortar elements. It is intended that the results of this research contribute to an increase in the reuse of this type of RAs.

## 2. Materials and Methods

### 2.1. Obtaining Recycled Aggregates

The RFAs used in this research were obtained from the renovation of the second floor of the “K” building of the Academic Division of Information Technology and Systems, at the Universidad Juárez Autónoma de Tabasco (UJAT, Cunduacán, Tabasco, Mexico). This two-story building is 18 years old. As part of the renovation, two separation walls made of prefabricated mortar elements (mortar blocks) were demolished. Since the first objective of this research was to determine the effect of using demolition strategies in obtaining the RAs, the demolition of one of the walls was planned and coordinated with the builder. Demolition of the second wall proceeded according to the original plan of the builder (conventional demolition). Therefore, two types of RA were obtained: the RFA*, obtained through a deconstruction process, and the RFA, produced through a conventional demolition.

The proposed deconstruction plan was simple and consisted of five steps: (1) estimation of the CDW volume to be obtained, (2) location of the rubble storage site, (3) removal of surface materials other than mortar (wood, metals, plastics, crystals, etc.) prior to demolition, (4) manual demolition followed by handling and separation of the remaining waste, and (5) crushing and storage. Because the ground floor of the building was empty due to the renovation work, it was used as a CDW warehouse. Before starting with the demolition of the walls, the window glass was removed, and the largest possible amount of paint was removed with a wire brush and spatulas. After the demolition process of the walls, the rubble was moved to the storage point (the ground floor of the building). Once all of the debris was deposited on the ground floor, personnel with safety equipment removed materials such as cables and some electrical devices that remained among the debris. The next step was to crush the rubble, thus obtaining the RFA*. For grinding, a Los Angeles abrasion machine was used. Subsequently, the debris was screened by means of an sieve number 4 (4.75 mm) and stored separately on the ground floor of the building, protecting it from the weather.

The demolition of the second separation wall produced the RFA. The conventional demolition process used by the builder was one that is commonly carried out in this type of remodeling, which consists of three stages: (1) removal of elements contemplated for reuse in the project (in this case none), (2) demolishing of the wall of prefabricated elements using hand tools, and (3) storage of all debris in an area away from the construction zone, to be later transferred to an authorized dumping area. For research purposes, debris from the separation wall was collected before being transferred to the municipal landfill. As in the previous case, before crushing and sieving with the number 4 sieve, materials other than mortar were removed as much as possible. The RFA obtained from this process was stored in a container in front of the building that was being renovated.

### 2.2. Characterization of the Materials

**Figure 1:**
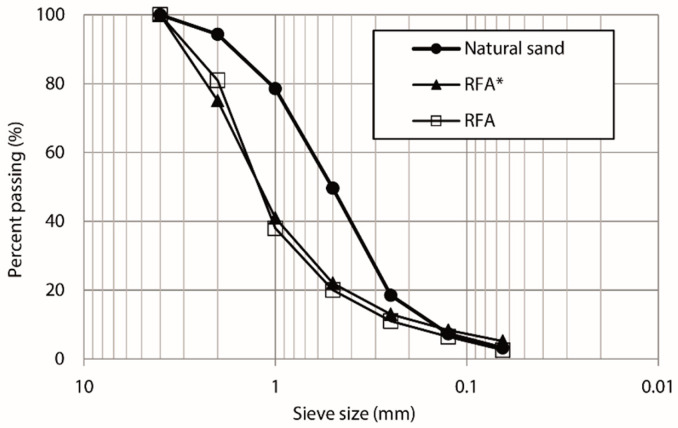
Particle size distribution of the sand and recycled aggregates.

All the RAs used in this research were characterized according to the UNE-EN 13139 [43] standard on mortar aggregates. Natural sand (NS) obtained from a riverbank was used as a reference element. The properties that were analyzed, as well as their reference standard, are shown in Table 1. Table 2 and Figure 1 show the particle size distribution of the sand and recycled aggregates. It was observed that in general, the granulometry of the aggregates obtained from prefabricated mortar pieces (RFA* and RFA) are similar.

The cement that was used is of the PCC 30R type Cemex^®^ brand (Monterrey, México), referring to a Portland cement compound of resistant class 30 with rapid resistance (3 days). This cement meets the international requirements of ASTM C150 / C150M-09 [44] and ASTM C595 / C595M-19 [45]. Table 3 shows its chemical characteristics.

### 2.3. Mixes

All mixtures were prepared with the same proportion and content of cement. A conventional mortar mixture (cement, natural sand, and water) was developed as a reference parameter. For the first phase of this investigation, two families of mixtures were prepared with the gradual replacement of NS with RA: RFA* was used in one and RFA in the other. For the second phase, two more types of mixtures were made. In all of them, the RFA* was taken as a substitute for sand. In one of them, the RFA* was subjected to pre-wetting before mixing (RFA* + h) and in the other, the RFA* was used with its natural moisture, but a commercial plasticizer was added to the mixture (RFA* + P) (Table 4). As a result of the above, five families of mortar were defined: NS, RFA*, RFA, RFA* + h, and RFA* + P.

Four replacement ratios were defined: 20%, 40%, 60%, and 100%. The replacement was carried out as a percentage of dry weight [19,50]. In total, 19 mixtures were made. Table 4 shows the nomenclature which was used.

The plasticizer which was used was Sikament 500, which is a medium-range water reducing liquid additive that does not contain chlorides. It complies with ASTM-C-494 Type D [51] and with ASTM-C-1017 Type II [52]. Its density is 1.20 ± 0.05 kg/L.

Mortar dosages were implemented according to the characteristics which were obtained for the materials. The following criteria were established:

- All the RAs that were used were smaller than 4 mm in particle diameter.
- The cement-aggregate ratio used in all mixtures was 1: 4.
- The amount of water was adjusted experimentally to achieve a consistency of 175 ± 5 mm in the mixtures.
- Pre-wetting of the aggregates was performed only in one type of mixture (RFA* + h). The rest of the aggregates were used with their natural humidity (2.3 ± 0.2). Pre-wetting was performed to reach 80% of the total absorption capacity of the RA, guaranteeing the presence of water in the aggregate and decreasing the migration of water from the mixture to the RA [5,41]. The procedure used to achieve the aforementioned wetting was based on that described by Fonseca et al. [53]: the aggregate was immersed in water for five minutes and then allowed to drain before its use.

The plasticizer was used in a proportion of 1% of cement weight. This value was recommended by the manufacturer. Table 5 shows mixture proportions used in this research. This table shows that according to the literature that was consulted [9,13,42], mortars containing RA need a larger amount of water to achieve project consistency.

The mixtures were made in a standard mixer, placing the cement and fine aggregate first, then mixing for a minute. Then, during the next 20 s, water was added while the mixer was still mixing the cement and aggregate. The mixing of these materials was continued for three minutes at a speed of 140 rpm. This procedure was followed for all mixtures to which no plasticizer was added. For the latter, we relied on the procedure described by Jiménez et al. [19]: water and additive were first placed in the mixer´s container, then mixed at low speed (140 rpm) for two minutes, after which cement and aggregate were slowly added. All these materials were mixed at low speed for three minutes.

### 2.4. Rehearsal Program

Assessing the properties of the mortar in its fresh state is an important aspect, because its characteristics in this state have a great impact on the performance of the hardened mortar. To evaluate the properties of fresh mortar, bulk density and air content tests were used. Whereas to characterize hardened mortar, dry bulk density, compressive strength, adhesive strength, and water absorption coefficient due to capillary action were tested. Table 6 shows the standards used during the tests.

## 3. Results and Discussion

### 3.1. Deconstruction Process Effect

Table 7 shows the results of the characterization of natural sand and recycled aggregates. The percentage of fine content refers to particles smaller than 0.063 mm. The sand equivalence values for recycled aggregates are similar to those of natural sand. According to the background literature review, recycled aggregates have a lower dry density than natural sand (NS). As expected, the water absorption value in all recycled aggregates is high with respect to that of NS [29,30]. The absorption of RFA is slightly higher than that exhibited by RFA*, probably due to the impurities that the first of these aggregates possesses.

All recycled aggregates meet with the specifications of the UNE-EN 13139 [43] standard for mortar aggregates. The quantity of sulfates (≤0.8), chlorides (≤0.15), and total sulfur (≤1) meet with the limits established by the UNE-EN 1744-1 standard [49]. Due to the characteristics of the processes through which the RAs were recovered, the presence of other contaminants that could change the properties of the mortars was not observed. However, it can be observed that although the total sulfate, chloride, and sulfur values satisfy the established quality standards, the contents of these substances in the RAs obtained through a conventional demolition (RFA) are a bit higher than those obtained through a deconstruction process (RFA*). This shows the benefit of planning a demolition according to the recycling of CDW.

### 3.2. Fresh Mortar

#### 3.2.1. Bulk Density of Fresh Mortar

**Figure 2:**
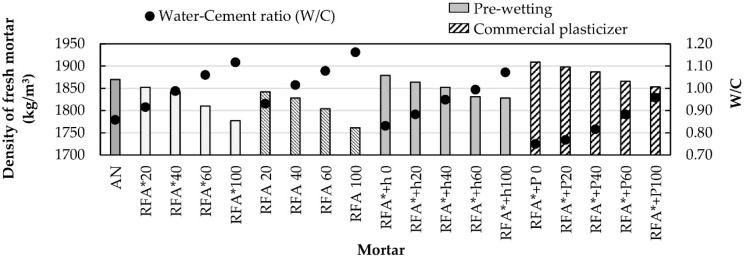
Bulk density of fresh mortar and W/C ratio.

Figure 2 shows the changes in bulk density of fresh mortar with respect to the percentages of substitution of natural sand (NS) with RA, as well as its corresponding W/C ratio.

It can be seen that relative to the control sample (NS), the density of mortars with RA decreases as the replacement ratio increases. This is a result of the low density of the RA and its high absorption [11]. The latter characteristic makes it necessary to increase the amount of water in the mixture to achieve project consistency (175 ± 5 mm). This increases the W/C ratio, thereby decreasing the density of the mixture. These results match those observed by other researchers [20,60]. The comparison of density values obtained for the families of RFA* and RFA mixtures, shows that the latter exhibit lower density for the same replacement ratios, as a consequence of their greater absorption.

On the other hand, the samples that used RFA* and underwent a pre-wetting process (RFA* + h), as well as those made with the commercial additive (RFA* + P), showed the highest densities for each of the replacement proportions. This increase in density is due to the fact that in these samples, the volume of free water was reduced in the mixture, allowing a larger quantity of solid particles in the mortar paste and therefore decreasing the W/C ratio.

#### 3.2.2. Air Content in Fresh Mortar

**Figure 3:**
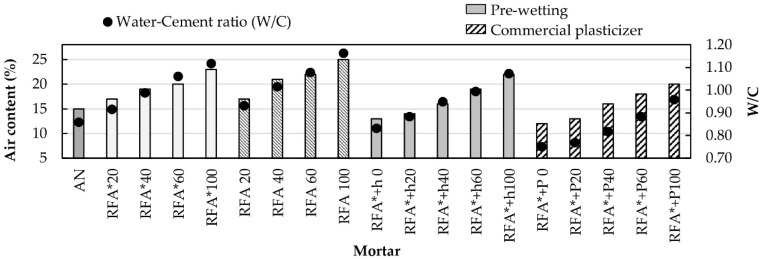
Air content of fresh mortar and W/C ratio.

Air content in mortar mixtures is an important parameter because it influences aspects such as durability and compressive strength. In spite of this, there are no specified limits for the amount of air in mortar mixtures. However, some researchers suggest that acceptable air contents are in the range of 5% to 20%, which was adopted as a reference in this work [40]. Figure 3 shows the variation of air content in fresh mortar with respect to the percentages of substitution of natural sand (NS) for RA, as well as its corresponding W/C ratio.

It is observed that the air content of the mortars manufactured with RFA* and RFA is greater than that of the control mixture, and also increases as the replacement ratio and the W/C ratio increase. In general, both families of mixtures show similar behavior, however, mixtures of the RFA family exhibit a slightly higher air content, due to their higher W/C ratio. On the other hand, the mixtures of the RFA* + w and RFA* + P families proved to have the lowest air content values, because they reduce the amount of water in the cement paste. Mixtures of the RFA* + P family showed the lowest air content values, all of them within the recommended ranges.

Figure 2 and Figure 3 clearly show the influence of the W/C ratio on the bulk density and on the air content of fresh mortar, respectively [19,40,61].

### 3.3. Hardened Mortar

#### 3.3.1. Dry Bulk Density

**Figure 4:**
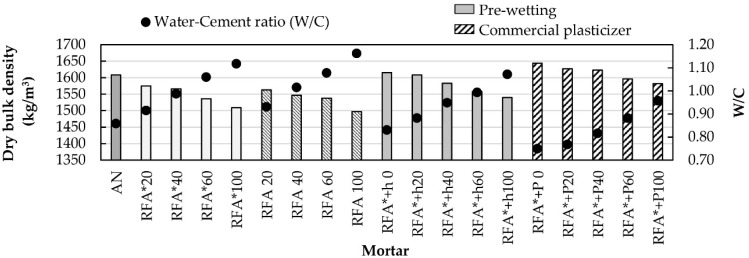
Dry bulk density and W/C ratio.

The bulk density of the hardened mortar at an age of 28 days and the W/C ratio for all mortar families are shown in Figure 4. As happens in the fresh state, it is observed that as the replacement percentage and the W/C ratio increase and density decreases, which is consistent with what was observed by other researchers [40]. It is noted that the control mortar (NS) develops a greater density than that of mortars of the RFA* and RFA families. An analysis among the latter makes it clear that mortars manufactured with aggregates obtained by a deconstruction process exhibit a higher dry bulk density, confirming the trends shown by the bulk density in the fresh state. Again, the best results were shown by mortars of the RFA* + h and RFA* + P families. The mixtures that incorporated commercial plasticizer exhibited the highest dry bulk densities for each percentage of substitution, in agreement with the observations of Pereira et al. [8] and Cartuxo et al. [11]. This is because these mixtures have the lowest values of W/C ratio and air content.

#### 3.3.2. Compressive Strength

**Figure 5:**
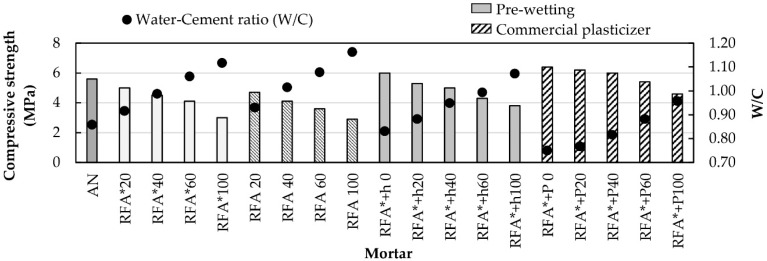
Compressive strength of the mortar at 28 days and W/C ratio.

Regarding the characteristics of mortar in the hardened state, compressive strength is the most important because it largely determines durability. Figure 5 shows the compressive strength at 28 days and the W/C ratio for all mortar families.

Continuing with the trend of previously analyzed properties, compressive strength decreases as the percentage of sand substitution by RA increases, as can be seen in Figure 5. This is in agreement with observations by Cuenca-Moyano et al. [40] and Zhao et al. [42]. The decrease in the compressive strength of all mixtures as the percentage of substitution increases is due to the increase in the W/C ratio required to maintain workability. Examining the compressive strengths of the RFA* and RFA mixture families, it can be observed that the former reach slightly higher values. These results confirm the importance of obtaining RA through a deconstruction process. The mixtures made with pre-wetting of the aggregates (RFA* + h) exhibited a greater resistance for each substitution level than the RFA* and RFA families, which is indicative of the importance of pre-wetting the aggregate before making the mortar mixtures. The highest resistances were obtained for mixtures added with commercial plasticizer. This is because this family of mixtures required less water during its manufacture, so the W/C ratio was lower. In Figure 5, it can be seen that the control mixture meets the minimum resistance indicated for M5 mortar (5 MPa). The RFA* and RFA mixtures did not meet this requirement. On the other hand, of the pre-moistened mixtures, only those with substitution percentages ranging from 60% to 100% were below the 5 MPa resistance. On the other hand, it can be seen that in mixtures with commercial plasticizer added, only those with a 100% substitution do not meet the minimum resistance value for the type of mortar used.

According to these results, it is possible to replace up to 40% of NS with RFA*, with 80% pre-wetting without affecting the compressive strength of the mortar. If commercial plasticizer is used, up to 60% of NS can be replaced with good results.

#### 3.3.3. Adhesive Strength

**Figure 6:**
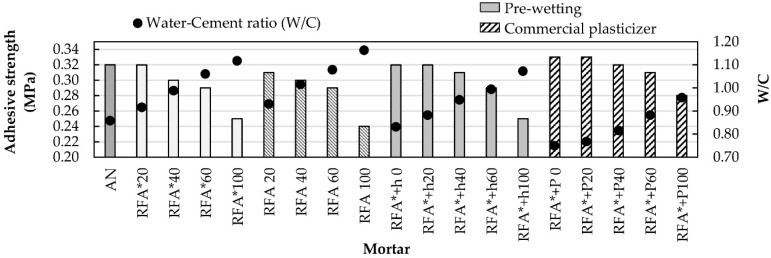
Mortar adhesion and W/C ratio.

Adhesive strength is a fundamental property of masonry mortars. The characterization of this property was performed using the European standard UNE-EN 1015-12 [58]. Figure 6 shows the results obtained for each of the families of mixtures. In general, the same trend is observed as for the other properties studied so far: the adhesive strength is affected as the replacement ratio and the W/C ratio increase. These observations are consistent with those made by Amorim et al. [62] and Silva et al. [18]. Comparing with mixtures to which no plasticizer was added, it is noted that similar behaviors exist for the same replacement rates. The highest adhesive strength values were obtained for mixtures added with commercial plasticizer.

One aspect to highlight is the fact that all mixtures with a 20% substitution percentage exhibit values similar to those of the reference mortar (NS). This seems to indicate that for this substitution percentage, there is no negative effect due to the incorporation of RA. On the other hand, Ledesma et al. [1], Neno et al. [13], Braga et al. [12], and Jiménez et al. [19] pointed out that using recycled fine concrete as a substitute for sand in a proportion between 10% and 20% increases adhesiveness with respect to mortar made with natural sand.

#### 3.3.4. Water Absorption Due to Capillary Action

Water absorption due to capillarity is an important indicator of mortar durability. It is known that high absorption values are related to less durability. If the mortar has high water absorption, it will allow the appearance of humidity, as well as water carried particles and substances detrimental to mortar durability. Water absorption due to capillary action depends on mortar structure. The more compact it is, the smaller the pore network is. Consequently, denser mortars will have less water absorption [63].

**Figure 7:**
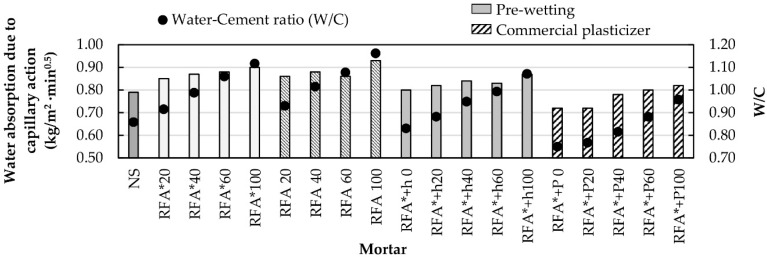
Water absorption values and W/C ratio.

Figure 7 shows water absorption values due to capillary action and water-cement ratio (W/C) for all the mixtures under observation. It is observed that mortar water absorption values increase as the W/C ratio and the NS replacement proportion by recycled aggregate increase [32,63]. As previously mentioned, if the amount of recycled aggregate is greater, the volume of water required will also be greater to maintain the consistency of the project (175 ± 5 mm), which increases the W/C ratio and consequently, mortars are created with a higher pore network that allows better water absorption.

RFA* + h and RFA* + P mixture families are shown to have the lowest absorption values due to their low water consumption. These mixtures exhibit a water consumption similar to the control mixture for substitution percentages of 20% and 60%, respectively. The mix made with the commercial additive and 20% RFA (RFA* + P20) exhibits less water absorption than the conventional mortar mix.

## 4. Conclusions

In this research, the use of RA obtained from mortar elements as a substitute for natural sand in the preparation of masonry mortars was evaluated, as a proposal of a new field of application for these RAs. Two types of recycled aggregates were used in this research: RFA* and RFA. However, mixtures developed using aggregates obtained through a process specifically planned to recover debris for its use as a substitute for sand (RFA*) developed higher bulk density, adhesive strength, and compressive strength for each substitution percentage than those made with aggregates obtained through conventional demolition (RFA). The deconstruction technique used in this study is simple and competitive in terms of cost, since the RA was obtained directly on-site and transport costs were reduced.

The experimental results show that the bulk densities in the fresh and hardened state, as well as the compressive and adhesive strengths of the mortars, all exhibit the same tendency: they decrease in an almost linear way with the rate of replacement of NS by RA. Regarding the air content and water absorption due to capillary action in the mixtures, it is clear that they increase as the content of RA and the W/C ratio increase. The main agent that causes these behaviors is the high absorption potential of the RAs. As the number of RA increases, it is necessary to add more water to the mixture to achieve project consistency. This is detrimental to the quality of the mortar. It is then concluded that the properties of the recycled mortar are closely linked to the substitution ratio of NS by RA and the W/C ratio.

The pre-wetting of RA at 80% of its absorption capacity before mixing prevents excessive water absorption by the cement paste. Therefore, mixtures that were made with this procedure (RFA* + h) showed an improvement of the analyzed properties, relative to mortar made with RA at natural moisture. The mixtures that were added to by a commercial plasticizer (RFA* + P), which required a smaller amount of water to achieve project consistency than the rest of the mixtures. This brought a considerable improvement of mortar properties, both in fresh and hardened states. Consequently, mortars with the greatest density, greatest strength, best adhesive strength and lowest absorption capacity were those added with plasticizer, followed by those made with pre-wetting of the aggregates. It was observed that, for the first of these two mixtures, a 60% substitution value produces very similar results to those obtained with the reference mortar, so this percentage was established as optimum. Concerning mortars with pre-wetting of the aggregate, the optimum replacement percentage was 40%. As it was demonstrated, these two techniques contribute to reducing the W/C ratio and improving the compacity and the mechanic properties of mortar, also avoiding the appearance of cracks produced by high quantities of free water.

Due to the fact that mortar mixtures made with both techniques have characteristics similar to those of conventional mortars (with their respective optimum percentage of substitution), it is probable that both kinds of mortars have a similar maintenance cost. However, more detailed studies about this topic are needed.

The experimental results show that it is possible to reuse RAs coming from prefabricated mortar elements if adequate debris recovery techniques are established and are used in combination with procedures that reduce the amount of water required in the mixtures.

The use of this type of materials in conventional applications of masonry mortar (indoors and outdoors) will be very important in the near future, since this practice is closely related to sustainability of construction technology.

## Figures and Tables

**Table 1:** Standards used in the characterization of natural sand and recycled aggregates.

Fine Content (%)	Sand Equivalent (%)	Dry Sample Density (gr/cm^3^)	Water Absorption (%)	Acid Soluble Sulphates (% SO_3_)	Water Soluble Chlorides (% Cl)	Total Sulphurs (% SO_3_)
UNE-EN 933-1 [46]	UNE-EN 933-8 [47]	UNE-EN 1097-6 [48]	UNE-EN 1097-6 [48]	UNE-EN 1744-1 [49]	UNE-EN 1744-1 [49]	UNE-EN 1744-1 [49]

**Table 2:** Particle size distribution.

Sieve Size (mm)		4	2	1	0.5	0.25	0.125	0.063
Percent passing(%)	NS	100	94	79	50	19	7.3	3.2
	RFA*	100	75	41	22	13	8.4	5.2
	RFA	100	81	38	20	11	6.5	2.6

**Table 3:** Chemical composition of cement (%) given by the fabricant, Cemex^®^.

Composition	CaO	SiO_2_	Al_2_O_3_	Fe_2_O_3_	MgO	K_2_O	Na_2_O	SO_3_
%	63	22	6	2.5	2.6	0.6	0.3	2.0

**Table 4:** Nomenclature for mortar mixes in which RA was used as a substitute of NS.

Replacement Ratio (%)	First Phase		Second Phase	
	RFA*	RFA	RFA* + h	RFA* + P
0	---	---	RFA* + h_0_	RFA* + P_0_
20	RFA*_20_	RFA_20_	RFA* + h_20_	RFA* + P_20_
40	RFA*_40_	RFA_40_	RFA* + h_40_	RFA* + P_40_
60	RFA*_60_	RFA_60_	RFA* + h_60_	RFA* + P_60_
100	RFA*_100_	RFA_100_	RFA* + h_100_	RFA* + P_100_

**Table 5:** Mortar mixture proportions.

Mortar Type	NS/RA	NS (gr)	RA (gr)	CEM (gr)	Water (gr)	Consistency Index (mm)	W/C
AN	100/0	2307	0	332	285	176	0.86
RFA*_20_	80/20	1845	462	332	304	172	0.92
RFA*_40_	60/40	1384	923	332	328	171	0.99
RFA*_60_	40/60	923	1384	332	352	177	1.06
RFA*_100_	0/100	0	2307	332	371	174	1.12
RFA _20_	80/20	1845	462	332	309	177	0.93
RFA _40_	60/40	1384	923	332	337	180	1.02
RFA _60_	40/60	923	1384	332	358	173	1.08
RFA _100_	0/100	0	2307	332	386	175	1.16
RFA* + h_0_	100/0	2307	0	332	276	170	0.83
RFA* + h_20_	80/20	1845	462	332	293	174	0.88
RFA* + h_40_	60/40	1384	923	332	315	179	0.95
RFA* + h_60_	40/60	923	1384	332	330	171	0.99
RFA* + h_100_	0/100	0	2307	332	356	176	1.07
RFA* + P_0_	100/0	2307	0	332	249	180	0.75
RFA* + P_20_	80/20	1845	462	332	255	173	0.77
RFA* + P_40_	60/40	1384	923	332	271	171	0.82
RFA* + P_60_	40/60	923	1384	332	293	172	0.88
RFA* + P_100_	0/100	0	2307	332	318	170	0.96

**Table 6:** Standards used in mortar characterization.

Test	Standard	Curing Time (Days)
*Properties of fresh mortar*		
Bulk density of the fresh mortar	UNE-EN 1015-6 [54]	---
Entrained air	UNE-EN 1015-7 [55]	---
*Properties of hardened mortar*		
Dry bulk density	UNE-EN 1015-10 [56]	28
Compressive strength	UNE-EN 1015-11 [57]	28
Adhesive strength	UNE-EN 1015-12 [58]	28
Water absorption coefficient due to capillary action	UNE-EN 1015-18 [59]	28

**Table 7:** Natural sand and recycled aggregate characterization.

Aggregate	Fine Content (%)	Sand Equivalent (%)	Dry Sample Density (gr/cm^3^)	Water Absorption (%)	Acid Soluble Sulphates (% SO_3_)	Water Soluble chlorides (% Cl)	Total Sulphurs (% SO_3_)
NS	9.12	95	2.65	0.28	<0.010	<0.010	<0.010
RFA*	4.12	84	2.1	6.76	0.0027	0.041	0.0027
RFA	6.56	86	1.98	7.22	0.0039	0.054	0.0039
